# Midline congenital upper lip sinus: a rare clinical case with analytical review of diagnostic and therapy strategies

**DOI:** 10.3389/fped.2026.1777126

**Published:** 2026-03-06

**Authors:** Shuang Yang, Kai Kang, Wei Liu, Zhibo Zhou

**Affiliations:** 1 National Engineering Laboratory for Digital and Material Technology of Stomatology, Beijing Key Laboratory of Digital Stomatology, Department of Oral and Maxillofacial Surgery, Peking University School and Hospital of Stomatology, Beijing, China; 2 The First Hospital of China National Pharmaceutical Group (Yiji Hospital), Baotou, Inner Mongolia, China

**Keywords:** congenital, malformation, midline, sinus, upper lip

## Abstract

Congenital midline sinus of the upper lip is rarest malformation with or without other anomalies. Mostly the sinus opens below the white role on the vermilion and has no intra-oral communication. To date, there have been only several case reports of upper lip sinuses associated with other anomalies, such as cleft palate or transverse facial cleft in China. We herein present a case of congenital upper lip sinus in the middle of the philtrum presenting as whitish discharge used to come out of it and review the current literature on this condition.

## Introduction

Congenital lip sinus, which may sometimes present merely as a superficial lip pit, is an exceptionally rare congenital malformation (1). Clinically, the lower lip sinus is the most commonly observed form, with a reported incidence of 0.001% in the general population (2). Its prevalence is even lower among Caucasians, approximately 0.00001%, while data on its occurrence in other ethnic groups remain scarcely documented, lower lip sinuses may occur in isolation or be associated with various syndromes, such as Van der Woude syndrome (3).

The incidence of upper lip sinus is even lower than that of lower lip sinus. The first case of congenital upper lip sinus was reported by Holbrook in 1970 (4). Clinically, the opening of an upper lip sinus is typically located in the philtrum, with the sinus tract directed toward the labial mucosa and usually not communicating with the oral cavity (5). Some literature has also reported that the blind end of the sinus tract may extend as far as the bony surface of the anterior nasal spine (6). Upper lip sinus may be associated with other midline anomalies, including double frenulum, frenulum sinus, nasal dermoid cyst, and hypertelorism (7). Due to its extremely low incidence and limited clinical reports, this article presents a Chinese pediatric case of congenital upper lip sinus (a rare subtype with ethnic and age characteristics) and reviews the existing literature to provide reference for clinical practitioners.

## Case report

An 8-year-old Chinese female patient initially presented to Baotou Guoyao Yiji Hospital (the Fourth Affiliated Hospital of Inner Mongolia Medical University, Baotou Stomatological Hospital) in July 2025, with the chief complaint of “intermittent liquid discharge from the upper lip upon compression for 8 years.”

Present Illness: The family reported that since birth, the child had experienced intermittent swelling of the upper lip. Pain or discomfort was denied. The swelling resolved after yellow-white non-odorous viscous secretions were expressed from the upper lip upon compression. No other discomforts were reported.

Past medical history was unremarkable, with no history of upper respiratory tract infection, oral soft tissue injury, or surgical intervention. There was no family history of congenital lip malformations, genetic diseases, trauma, or systemic illness.

### Specialized examination

**Figure 1:**
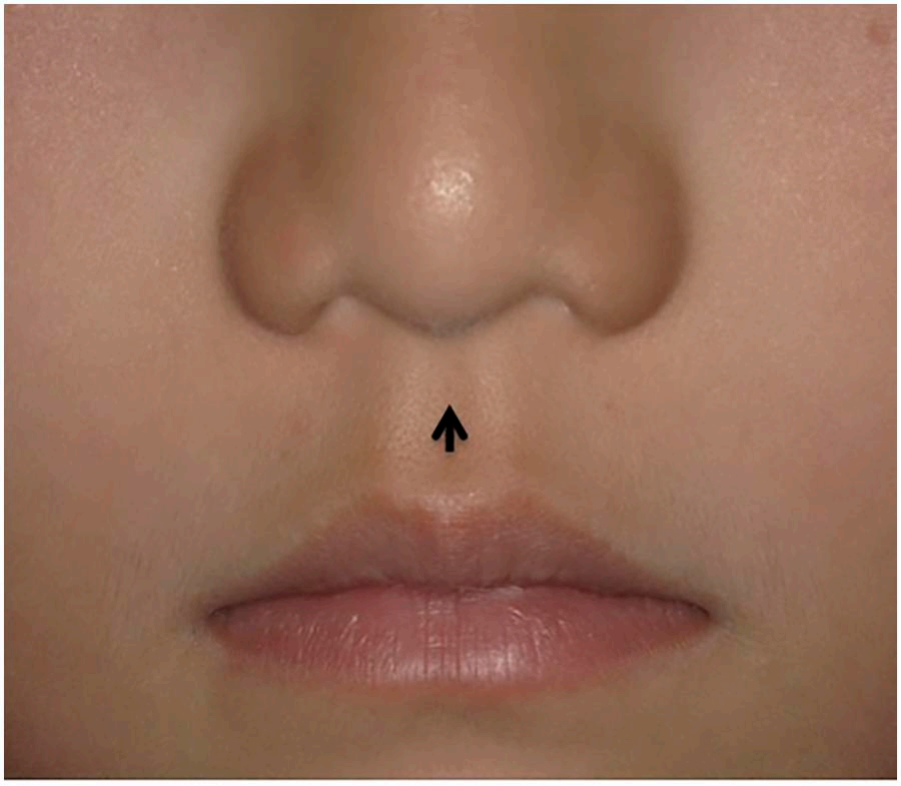
A dimper in the upper philtrum.

**Figure 2:**
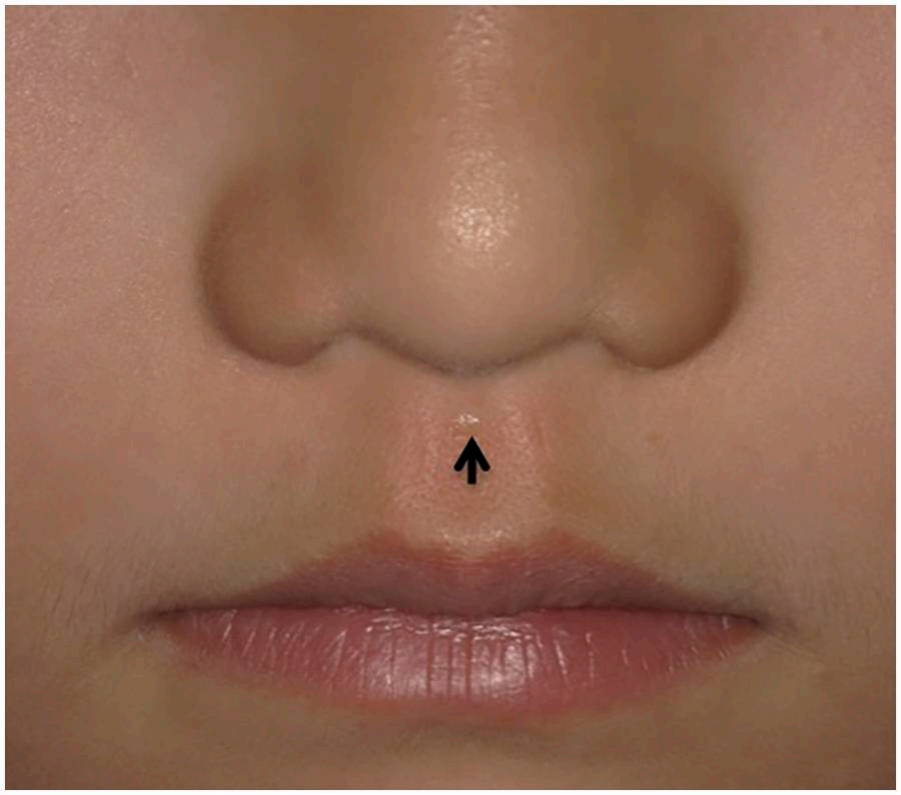
Discharge of white fluid though the orifice.

Clinical examination revealed a symmetrical facial contour, normal mouth opening, and no significant abnormalities in the bilateral temporomandibular joints. A sinus tract opening, approximately 0.5 mm in diameter, was observed at the philtrum near the nasal columella (Figure 1). The surrounding skin exhibited normal color and texture without significant abnormalities, and yellow-white discharge could be expressed upon compression (Figure 2). No fistula opening was found on the vermilion mucosa of the upper lip. The lower lip mucosa appeared normal with no sinus openings. The upper and lower dental arches were aligned, with no evidence of caries in the anterior maxillary teeth. The palate was intact without clefting, and the labial and buccal frenula were normal.

### Diagnosis

Congenital Upper Lip Sinus, Aoki Type I

### Treatment plan

Given the patient's absence of a history of recurrent infections, no cosmetic impairment, and no functional disturbance (eating/speaking/breathing), a conservative watchful waiting strategy is currently recommended. Specific follow-up suggestions: 1) Regular follow-up every 6 months in the stomatological outpatient department, including clinical examination and ultrasound reexamination; 2) Family education, such as, keeping the local skin clean, and seeking medical attention immediately if symptoms such as redness, swelling, pain, or increased secretions occur (suggesting infection); 3) Surgical excision under general anesthesia (pediatric-specific anesthesia scheme) is recommended if recurrent infections, cosmetic dissatisfaction, or functional impairment develop in the future.

## Discussion

Congenital upper lip sinus typically presents as a sinus tract in the midline of the upper lip, though paramedian locations are also possible. The sinus opening may be situated on either the vermilion mucosa or the cutaneous surface of the upper lip, extending deep into the orbicularis oris muscle and usually not communicating with the oral cavity (7). The sinus opening typically measures approximately 1–2 mm in diameter, while the tract length ranges from about 5 to 30 mm (8).

Upper lip sinus is a rare condition. A review of the existing English literature identified 69 reported cases, as summarized in Supplementary Table S1.

Geographically, 36 cases (52%, 36/69) were reported in Asia, of which only 3 cases were from China—our case is the first detailed pediatric case report of congenital midline upper lip sinus in North China (Inner Mongolia), filling the gap in epidemiological and clinical data of this disease in northern Chinese pediatric populations. In terms of gender distribution, 83% of Aoki Type I cases are female, which is consistent with our female patient, further confirming the significant female predilection of this subtype. In terms of age at diagnosis, most existing reports are in adults, and pediatric cases (≤12 years old) account for less than 15% of the total cases, indicating that pediatric upper lip sinus is easily overlooked in clinical practice due to mild symptoms—our case highlights the importance of early clinical identification in pediatric patients with intermittent lip discharge since birth.

Aoki (2011) summarized 31 cases of upper lip sinus and classified them into three types: Type I (13 cases) involved only a midline sinus in the upper lip without associated anomalies; Type II (9 cases) consisted of a midline upper lip sinus accompanied by other anomalies; and Type III (9 cases) featured a sinus located on the lateral aspect of the lip, with or without other anomalies (9). Additionally, Aoki noted that among the 13 cases of Type I, 12 were female patients, indicating a significantly higher prevalence in females compared to males (9).

Three main theories have been proposed regarding the pathogenesis of the upper lip sinus. The first is the invagination theory, which suggests that it results from failed invagination of the ectoderm of the nasal placodes during the development of the frontonasal process (8, 10, 11). The second is the merging theory, attributing the condition to abnormalities in the normal fusion process of mesoderm (67). The third is the fusion theory, which proposes that it is caused by incomplete fusion between the frontonasal process and the maxillary process (12, 13, 68). However, the exact underlying mechanism for the formation of the upper lip sinus remains unclear. Notably, no associated midline anomalies were found in our case, which supports the invagination theory (a single ectodermal developmental abnormality without mesodermal involvement)—this finding provides a small sample clinical evidence for the pathogenesis research of Aoki Type I upper lip sinus, supplementing the existing etiological data.

Clinically, patients with an upper lip sinus often present with recurrent swelling and discharge of fluid from the sinus opening upon compression. Diagnosis is typically established through specialized clinical examination, supplemented by auxiliary imaging modalities such as ultrasonography and spiral computed tomography (CT). On ultrasound, the sinus appears as a hypoechoic tract extending from the skin towards the orbicularis oris muscle, while spiral CT reveals a corresponding hypodense channel⁷. Approximately 25% of patients experience symptoms of recurrent infection (7, 14).

For cases with recurrent infections, the primary treatment is surgical intervention, which involves complete excision of the sinus tract. Incomplete resection may lead to postoperative swelling and persistent infection. The patient reported in this study had a history of swelling and discharge from the sinus opening but no documented episodes of infection. Therefore, a watchful waiting approach was recommended, with surgical intervention advised only if recurrent infections occur.

The therapeutic management of congenital upper lip sinus is controversial in existing literature, with no unified criteria for choosing conservative treatment or surgical intervention. Based on our case and analytical review of 69 reported cases, we propose individualized therapeutic management principles based on symptoms, age, and clinical subtype:

Conservative watchful waiting: indicated for patients with no recurrent infections, no cosmetic/functional impairment (regardless of age), especially pediatric patients (avoid unnecessary general anesthesia and surgical trauma); regular follow-up and local care are the key, as observed in our case.
Surgical excision: indicated for patients with recurrent infections, cosmetic dissatisfaction, or functional disturbance; the surgical approach should be selected based on the Aoki type: Type I/II (midline)—intraoral mucosal incision (preferred, avoid cutaneous scar, suitable for cosmetic demands such as children and young adults); Type III (lateral)—direct opening incision (feasible, easy to expose the sinus tract). The surgical approach varies slightly depending on the classification. For patients with Aoki Type I and II sinuses, where the orifice is located in the midline skin of the upper lip, previous literature describes an intraoral mucosal incision to completely excise the fistula, thereby avoiding a cutaneous scar that could affect aesthetics (14). Alternatively, other reports advocate for a direct skin incision for complete resection (15, 16). For Aoki Type III sinuses, with the orifice situated on the lateral lip mucosa, a direct incision at the sinus opening is feasible for complete excision (17). Postoperative histopathological examination typically reveals that the sinus tract is lined by well-differentiated stratified squamous epithelium and is surrounded by skin, subcutaneous tissue, and muscle layers (15).
Pediatric-specific considerations: general anesthesia is required for pediatric surgical patients, and the operation should be delayed as much as possible until the age of 6–12 years (when facial development is relatively mature) if there is no urgent indication, to reduce the impact of surgery on facial development.

## Summary

Congenital midline upper lip sinus is an extremely rare congenital malformation, with pediatric cases featuring unique clinical characteristics (pinpoint-like opening, short sinus tract) that are easily overlooked in clinical practice. This study presents the first detailed pediatric case of congenital midline upper lip sinus in North China (Inner Mongolia), which enriches the epidemiological and clinical data of this disease in northern Chinese pediatric populations. Through an analytical review of existing literature, we propose a practical stepwise diagnostic strategy (ultrasound as the first-line imaging modality for pediatric patients) and individualized therapeutic management principles (based on symptoms/age/subtype) for congenital upper lip sinus, which clarify the controversial diagnostic and therapeutic criteria in existing literature and provide a more practical reference for clinical practitioners in pediatric, stomatological, and oral and maxillofacial surgery departments. The limitations of this study are the single case report, and multi-center, large-sample clinical studies are needed in the future to further verify the proposed diagnostic and therapeutic strategies.
