# Supplementary material for: Midline congenital upper lip sinus: a rare clinical case with analytical review of diagnostic and therapy strategies
Source: Front Pediatr. 2026 Mar 6;14:1777126. doi: 10.3389/fped.2026.1777126 (PMC13003570; doi:10.3389/fped.2026.1777126)
Supplement: Supplementary file 1 [file Supplementaryfile1.pdf]

References

1 Charrier, J. B., Rouillon, I., Roger, G. *et al.* Congenital isolated midline sinus of the upper lip: clinical and embryological approaches [Journal Article]. *Cleft Palate Craniofac J* 2006,4(**43**): 488-491.

2 Sancho, M. A., Albert, A., Cusi, V. *et al.* Upper lip fistulas: three new cases [Journal Article]. *Cleft Palate Craniofac J* 2002,4(**39**): 457-460.

3 Cervenka, J., Gorlin, R. J. & Anderson, V. E. The syndrome of pits of the lower lip and cleft lip and/or palate. Genetic considerations [Journal Article]. *Am J Hum Genet* 1967,3 Pt 2(**19**): 416-432.

4 Holbrook, L. A. Congenital midline sinus of the upper lip [Journal Article]. *Br J Plast Surg* 1970,2(**23**): 155-160.

5 Salati, S. A. & Al Aithan, B. Congenital median upper lip fistula [Journal Article]. *APSP J Case Rep* 2012,2(**3**): 11.

6 Rokaha, P. S., Paudel, S., Jha, A. *et al.* Infected type 1 congenital upper lip sinus in 8 years child: A rare case report [Journal Article]. *Radiol Case Rep* 2025,9(**20**): 4551-4554.

7 Hili, S., Wong, K. Y. & Goodacre, T. Isolated midline upper lip pit [Journal Article]. *BMJ Case Rep* 2016,**2016**): 10 1136/bcr-2016-215496.

8 Pitanguy, I. & Franco, T. Nonoperated facial fissures in adults [Journal Article]. *Plast Reconstr Surg* 1967,6(**39**): 569-577.

9 OR., M. n. Congenital pit of the upper lip. [Journal Article]. *Oral Surg Oral Med Oral Pathol.* 1969,**27**): 441-444.

10 Mackenzie KI. Mid-line sinus of the upper lip. J Laryngol Otol 1970; 84:235-8. [Journal Article].

11 Bartels, R. J. & Howard, R. C. Congenital midline sinus of the upper lip. Case report [Journal Article]. *Plast Reconstr Surg* 1973,6(**52**): 665-668.

12 Parisier, S. C. & Birken, E. A. Congenital midline sinus of the upper lip [Journal Article]. *Arch Otolaryngol* 1973,3(**97**): 259-262.

13 Kriens O, Schmidt H, Mueller-Driver O. Congenital lateral stula of upper lip. A case report. J Maxillofac Surg 1973; 1:122-4. [Journal Article].

14 Mahler, D. M. & Karev, A. Lateral congenital sinus of the upper lip [Journal Article]. *Br J Plast Surg* 1975,3(**28**): 203-204.

15 Miller, C. J. & Smith, J. M. Midline sinus of the upper lip and a theory concerning etiology [Journal Article]. *Plast Reconstr Surg* 1980,5(**65**): 674-675.

16 Hosokawa, K., Susuki, T., Kikui, T. A. *et al.* A congenital lateral sinus in the upper lip [Journal Article]. *Ann Plast Surg* 1983,1(**11**): 69-70.

17 Urade, M., Tofani, I., Igarashi, T. *et al.* Congenital midline sinus of the upper lip: report of a case [Journal Article]. *J Osaka Univ Dent Sch* 1984,**24**): 59-65.

18 Grenman, R., Salo, H. & Rintala, A. Midline sinuses of the upper lip. Case report [Journal Article]. *Scand J Plast Reconstr Surg* 1985,2(**19**): 215-219.

19 Raibagkar S, V. U. Lateral congenital sinus of the upper lip. [Journal

Article]. *Indian J Plast Surg* 1986,50-52(**19**.
20 Galderon S, G. J. Surgical excision of a congenital lateral fistula of the upper lip. [Journal Article]. *J Craniomaxillofac Surg.* 1988,**16**): 46-48.
21 Katou, F. & Motegi, K. Congenital midline sinus of the upper lip. Report of a case [Journal Article]. *International journal of oral and maxillofacial surgery* 1989,4(**18**): 237-238.
22 Y., T. Congenital lateral fistula of the upper lip. [Journal Article]. *J Craniomaxillofac Surg.* 1989,**17**): 186-189.
23 Sakamoto, H., Imai, Y. & Asakura, A. Congenital midline sinus of the upper lip. Report of a case [Journal Article]. *International journal of oral and maxillofacial surgery* 1992,1(**21**): 10-11.
24 Eppley, B. L., Sadove, A. M. & Goldenberg, J. Philtral fistula in median cleft lip: cause and effect or coincidence? [Journal Article]. *Ann Plast Surg* 1992,3(**29**): 263-265.
25 Mizuki, H., Shimizu, M. & Danjo, T. Congenital fistula with an island of vermilion-epithelium in the paramidline of the upper lip: report of a case [Journal Article]. *Journal of cranio-maxillo-facial surgery : official publication of the European Association for Cranio-Maxillo-Facial Surgery* 1993,1(**21**): 22-24.
26 Asahina, I., Sakakibara, T., Miyashin, M. *et al.* Congenital midline sinus of the upper lip: case report and review of literature [Journal Article]. *Cleft Palate Craniofac J* 1997,1(**34**): 83-85.
27 Y. Shigihara, M. N. Midline sinus of the upper lip associated with an intraoral polyp. [Journal Article]. *Eur. J. Plast. Surg.* 1997,**21**): 151-153.
28 Licht, A., Posner, J. & Leipziger, L. S. Median cleft lip with associated midline sinuses [Journal Article]. *J Craniofac Surg* 1998,4(**9**): 366-370.
29 Rifaat M, J. I., Celebilor O. The lateral upper lip sinus: case report and review of literature. [Journal Article]. *Eur J Plast Surg.* 1998,**21**): 98.
30 Illing, H. M., Field, D., McNamara, C. M. *et al.* Congenital sinus of the upper lip. A case report [Journal Article]. *International journal of oral and maxillofacial surgery* 1999,1(**28**): 29-30.
31 Al-Qattan, M. M. Congenital midline sinus of the upper lip [Journal Article]. *Ann Plast Surg* 2000,1(**44**): 76-78.
32 Nagasao T, U. S., Yokoi K, Yotsuyanagi T, Sawada Y. . A case of congenital midline sinus of the upper lip. [Journal Article]. *Eur J Plast Surg.* 2000,**23**): 289–293.
33 Ozgur, F. & Tuncbilek, G. Bilateral congenital pits of the upper lip [Journal Article]. *Ann Plast Surg* 2000,6(**45**): 658-661.
34 Sumitomo, S., Ogawa, S., Hirai, K. *et al.* Congenital sinus of the upper lip with idiopathic precocious puberty [Journal Article]. *Oral diseases* 2002,6(**8**): 308-309.
35 Lee TJ, B. C., Koh CS. Congenital sinus of upper lip. [Journal Article]. *J Korean Cleft Palate Craniofac Assoc* 2003,**4**): 45.
36 Sen, C., Agir, H., Isken, T. *et al.* Congenital midline upper lip sinus [Journal

Article]. *J Craniofac Surg* 2006,4(**17**): 810-811.
37 Tuncali, D., Barutcu, A. Y., Terzioglu, A. *et al.* Two cases of congenital midline upper lip sinuses associated with miscellaneous deformities [Journal Article]. *Journal of oral and maxillofacial surgery : official journal of the American Association of Oral and Maxillofacial Surgeons* 2006,4(**64**): 734-737.
38 Nakano, Y., Somiya, H., Shibui, T. *et al.* A case of congenital midline fistula of the upper lip [Journal Article]. *Bull Tokyo Dent Coll* 2010,1(**51**): 31-34.
39 Aoki, M., Sakamoto, Y., Nagasao, T. *et al.* Classification of congenital midline upper lip sinuses: a case report and review of the literature [Journal Article]. *Cleft Palate Craniofac J* 2014,2(**51**): 154-157.
40 H. Jung, S. K. Congenital upper lip sinus found in adolescent patient: a case report. [Journal Article]. *Arch. Craniofac. Surg.* 2012,1(**13**): 57-59.
41 Bhatnagar, A., Musa, O., Gildiyal, J. P. *et al.* Congenital lateral upper lip sinus: a rare case [Journal Article]. *Indian J Surg* 2012,4(**74**): 344-347.
42 Fok, D., Kua, E. H. & Por, Y. C. Congenital midline sinus of the upper lip [Journal Article]. *Singapore Med J* 2015,6(**56**): e107-109.
43 Anicete RC, K. I., Tan HKK Congenital midline upper lip sinus: a case report. [Journal Article]. *Int J Pediatr Otorhinolaryngol* 2014,**9**): 18.
44 Bakshi, S. S., Kiruba Shankar, M. & Gopalakrishnan, S. Midline upper lip sinus: a case report and review of literature [Journal Article]. *Oral and maxillofacial surgery* 2015,2(**19**): 217-219.
45 James L, V. B., Ghouse N Congenital upper lip pit: a rare case report. [Journal Article]. *J Indian Acad Oral Med Radiol* 2015,**27**): 311.
46 Rohart, J., Nicot, R., Myon, L. *et al.* [Labial recurrent swelling revealing median congenital upper-lip fistula] [Journal Article]. *Rev Stomatol Chir Maxillofac Chir Orale* 2015,5(**116**): 315-319.
47 Xu, Y., Chen, R., Mu, Y. *et al.* Congenital Midline Sinus of the Upper Lip [Journal Article]. *J Craniofac Surg* 2016,5(**27**): 1306-1307.
48 Salah, B. I., Al-Rawashdeh, B., Al-Ali, Z. R. *et al.* Congenital midline sinus of the upper lip: A case report and review of literature [Journal Article]. *Int J Surg Case Rep* 2018,**51**): 41-44.
49 Alam, M., Hasan, S. A., Hashmi, S. F. *et al.* Isolated Congenital Midline Upper Lip Sinus in A 5-year Old Child: A Rarity [Journal Article]. *Indian J Otolaryngol Head Neck Surg* 2017,4(**69**): 568-570.
50 Kun-Darbois, J. D., Chatellier, A., Pare, A. *et al.* Congenital Midline Upper Lip Sinuses: 3 Rare Cases [Journal Article]. *Cleft Palate Craniofac J* 2018,2(**55**): 292-295.
51 Chowdhary, F., Chowdhary, A., Chowdhary, Z. *et al.* Midline Congenital Upper Lip Sinus: A Rare Case [Journal Article]. *Indian J Otolaryngol Head Neck Surg* 2019,Suppl 1(**71**): 553-557.
52 Hosokawa, T., Takahashi, H., Miyasaka, Y. *et al.* Ultrasound Evaluation of Dermal Sinuses/Fistulas in Pediatric Patients [Journal Article]. *J Ultrasound Med* 2019,12(**38**): 3107-3122.

53 Donnell, C. C., McKeague, K. F., Cooper, A. *et al.* Nasofrontal dermoid cyst: rare presentation of a philtrum sinus [Journal Article]. *The British journal of oral & maxillofacial surgery* 2020,5(**58**): 608-610.
54 Akatsuka, T., Omatsu, J., Miyagawa, T. *et al.* Congenital midline sinus of the upper lip: Evaluating the use of ultrasonography [Journal Article]. *Skin Health Dis* 2023,1(**3**): e170.
55 Kotowski, M. & Szydlowski, J. Congenital midline upper lip sinuses with intracranial extension - A variant of nasal dermoid? An embryology-based concept [Journal Article]. *Int J Pediatr Otorhinolaryngol* 2023,**164**): 111394.
56 Li, P., Wang, J. & Lin, L. Congenital Midline Upper Lip Sinus: Case Report and Review of the Literature [Journal Article]. *Ear Nose Throat J* 2024): 1455613241249052.
57 Zhang, J., Li, Z. & Pang, P. Congenital Upper Lip Sinus [Journal Article]. *J Craniofac Surg* 2024,5(**35**): e438-e441.
58 Jung, C., Gall, E. K. & Scott, A. R. The Role of Preoperative Imaging for Midline Congenital Upper Lip Sinus Tracts [Journal Article]. *Ann Otol Rhinol Laryngol* 2024,10(**133**): 902-909.
59 Sahu, A., Mahalik, S. K. & Tripathy, T. P. Congenital midline upper lip sinus in an infant [Journal Article]. *BMJ Case Rep* 2024,3(**17**.
60 Ichikawa, C., Takiguchi, T., Kakinuma, S. *et al.* A Case of Congenital Midline Upper Lip Fistula [Journal Article]. *J Plast Reconstr Surg* 2025,3(**4**): 164-168.
61 Parisier SC, Birken EA. Congenital midline sinus of the upper lip. Arch Otolaryngol 1973; 97:259-62. [J]",
62 Bartels RJ, Howard RC. Congenital midline sinus of the upper lip. Case report. Plast Reconstr Surg 1973; 52:665-8. [J]."

Table 1

	Author	Year	Case No.	Country	Site
1	Pitanguy et al.[8]	1967	1	?	lateral
2	Mene´ndez et al.[9]	1969	1	Guatemala	Midline
3	Holbrook et al.[4]	1970	1	America	Midline
4	Mackenzie et al.[10]	1970	1	America	Midline
5	Bartels et al.[11]	1973	1	?	Midline
6	Parisier et al.[12]	1973	1	?	Midline
7	Kriens et al.[13]	1973	1	Germany	Lateral
8	Mahler et al.[14]	1975	1	?	Lateral
9	Miller et al.[15]	1980	1	?	Midline
10	Hosokawa et al.[16]	1983	1	?	Lateral
11	Urade et al.[17]	1984	1	Japan	Midline
12	Grenman et al.[18]	1985	2	?	Midline
13	Raibagkar et al.[19]	1986	1	India	Lateral

14	Galderon et al.[20]	1988	1	?	Lateral
15	Katou et al.[21]	1989	1	Japan	Midline
16	Takenoshita et al.[22]	1989	1	?	Lateral
17	Sakamoto et al.[23]	1992	1	Japan	Midline
18	Eppley et al.[24]	1992	1	?	Midline
19	Mizuki et al.[25]	1993	2	Japan	Midline
20	Asahina et al.[26]	1997	1	Japan	Midline
21	Shigihara [27]	1997	1	Japan	Midline
22	Licht et al.[28]	1998	1	America	Midline
23	Rifaat et al.[29]	1998	1	America	Lateral
24	Illing et al.[30]	1999	1	United Kingdom	Midline
25	Al-Qattan et al.[31]	2000	1	Saudi Arabia	Midline
26	Nagasao et al.[32]	2000	1	Japan	Midline
27	Ozgur et al.[33]	2000	1	Turkey	Lateral
28	Sumitomo et al.[34]	2002	1	Japan	Midline
29	Sancho et al.[2]	2002	3	Spain	Midline
30	Charrier et al.[1]	2002	1	France	Midline
31	Lee et al.[35]	2003	1	Korea	?
32	Sen et al.[36]	2006	1	Turkey	Midline
33	Tuncali et al.[37]	2006	2	Turkey	Midline
34	Nakano et al.[38]	2010	1	Japan	Midline
35	Aoki et al.[39]	2012	1	Japan	Midline
36	Salati et al.[5]	2012	1	Saudi Arabia	Midline
37	Jung et al.[40]	2012	1	Korea	Midline
38	Bhatnagar et al.[41]	2012	1	India	Lateral
39	Fok et al.[42]	2014	1	Singapore	Midline
40	Anicete et al.[43]	2014	1	Singapore	Midline
41	Bakshi et al.[44]	2015	1	India	Midline
42	James et al.[45]	2015	1	India	Lateral
43	Rohart et al.[46]	2015	1	France	Midline
44	Hili et al.[7]	2016	1	United Kingdom	Midline
45	Xu et al.[47]	2016	1	China	Midline
46	Salah et al.[48]	2016	1	Jordan	Midline
47	Alam et al.[49]	2017	1	India	Midline
48	Kun-Darbois et al.[50]	2018	3	France	Midline
49	Chowdhary et al.[51]	2018	1	India	Midline
51	Takahiro et al.[52]	2019	1	Japan	Midline
52	Donnell et al.[53]	2020	1	United Kingdom	Midline
53	Akatsuka et al.[54]	2022	1	Japan	Midline
54	Kotowski et al.[55]	2023	2	Poland	Midline
55	Peng et al.[56]	2024	1	China	Midline
56	Zhang et al.[57]	2024	1	China	Midline
57	Jung et al.[58]	2024	3	America	Midline

58	Saku et al.[59]	2024	1	India	Midline
59	Ichikawa et al.[60]	2024	1	Japan	Midline
60	Rokaha et al.[7]	2025	1	Nepal	Midline
Total			69		
